# The relationship between ethnicity and place of birth in England: a mixed-methods study

**DOI:** 10.1186/s12884-024-06977-z

**Published:** 2024-11-27

**Authors:** Frances Rivers, Christopher Grollman, Zahra Khan, Marina A. S. Daniele

**Affiliations:** 1 Kingston Hospital NHS Foundation Trust, Galsworthy Road, Kingston Upon Thames, KT2 7QB England; 2 School of Health and Psychological Sciences, City, University of London, London, EC1V 0HB England

**Keywords:** Birthplace, Midwife-led unit, Birth centre, Homebirth, Ethnic minority, South Asian, Ethnicity

## Abstract

**Background:**

UK maternity policy advocates a choice of birthplace in an obstetric-led unit (OU), a midwife-led unit (MLU) or at home. Although robust evidence supports the safety of birth in midwife-led settings, particularly for women with uncomplicated pregnancies, most births are in the OU. Women and babies from ethnic minority communities experience major health disparities and inequitable care, but there is limited research examining birthplace choices through an ethnicity lens. This study investigated the association between ethnicity and place of birth at an urban NHS Trust in England.

**Methods:**

A mixed-methods sequential explanatory study. Analysis of births from 2014–2023 at a London NHS Trust included multivariable logistic regression analysis of birthplace by ethnicity. Planned or pre-labour Caesareans, pre-term, and multiple births were excluded. Significant disparities between White and South Asian women were identified which informed the focus of the qualitative study. Semi-structured interviews with 10 women of South Asian heritage who had given birth in the OU, the alongside MLU or at home were conducted and analysed thematically.

**Results:**

More White women gave birth in midwife-led settings (27.5%) than all other ethnicities, particularly South Asian women (20.6%). South Asian women had fewer homebirths (0.8%) than White women (2.7%) and were much less likely to birth in a midwife-led setting after adjusting for parity, maternal age, BMI, previous Caesarean, presence of diabetes or hypertensive disorders and onset of labour (aOR 0.61, 95% CI 0.51–0.73, *p* < 0.001). Places of birth were similar for Black and White women, although the number of Black women in the population was too low to detect significant differences.

Themes generated from interviews included the assumption that birth is hospital-based and doctor-led; choosing a midwife-led birth setting went against the cultural norm, but felt safe – physically, psychologically and culturally.

**Conclusions:**

There are ethnic disparities in place of birth. Cultural factors seem influential, but barriers to choice, such as limited evidence-sharing by midwives, may disproportionately affect women from ethnic minority communities, who may particularly benefit from midwife-led birth settings. Women need personalised information about options. Improving choice of birthplace is a step towards reducing health inequalities and promoting optimal health.

## Background

United Kingdom (UK) maternity guidelines and policies have long supported choice of birthplace, with the expectation that NHS Trusts should provide women with the option to give birth either in an obstetric-led unit (OU), a midwife-led unit (MLU) or at home [1–3]. Midwives have lead responsibility for the care provided in an MLU or at a homebirth, whereas obstetricians are accountable for the care in an OU.

A wealth of evidence supports the safety of midwife-led settings, particularly for women with uncomplicated pregnancies [4–9]. They provide holistic care that values choice and autonomy in an environment that supports physiological birth without unnecessary intervention. Systematic reviews of uncomplicated pregnancies in high-income countries have demonstrated no significant differences in neonatal outcomes by place of birth, including stillbirth and neonatal mortality [4, 5]. Mothers are less likely to have a Caesarean birth, instrumental vaginal birth, episiotomy, or severe perineal tear with no significant difference in postpartum haemorrhage rates [4, 7]. Midwife-led care is also highly valued by women who use it [10, 11].

Despite its well-documented benefits and evidence that more than one-third of women could birth in MLUs based on their risk profile at the onset of labour [12], only approximately 15–18% of women in England give birth in a midwife-led setting, including 2.5% at home [13, 14]. Influential factors include information “gatekeeping” by health professionals, lack of midwifery continuity, limited discussion time, views of friends and family, previous experiences, and beliefs about safety, transfer and pain relief [15–17]. In the national Maternity Survey of women’s experiences of pregnancy, 18% of respondents said they were not given any choice about birthplace, and 19% said they were not given enough information to help them decide where to give birth [18].

The landmark Birthplace in England study [19], provided comprehensive analysis of where women without medical or obstetric complexities choose to give birth. Maternal ethnicity did not alter the benefits of midwife-led care, including reduced rates of instrumental birth and intrapartum Caesarean, nor did it affect rates of transfer to obstetric-led care [20]. However, women planning to give birth at home or in a “freestanding” MLU (geographically separate from the OU) were more likely to be White and from a higher socioeconomic status. Whereas women planning birth in the OU were 82% White, 7% South Asian and 5% Black, those planning homebirths were 95% White and only 0.7% South Asian and 1.5% Black. The ethnicity of women planning birth in an “alongside” MLU (located on the same site as the OU) was similar to that of women in the OU group [19].

Since its publication in 2011, there has been limited research on the sociodemographic characteristics of women by place of birth and the extent to which women’s choices about, use of, or access to different places of birth are affected by their ethnicity. Studies have revealed incidental findings about ethnicity and place of birth which include women from ethnic minorities reporting less choice [21] and knowledge [22] about place of birth but experiencing high rates of midwife-led births if a midwifery continuity of carer model is implemented [23].

Women and babies from ethnic minority communities experience significant health disparities [24–26]. Maternal mortality rates are almost 4 times greater for Black women and almost 2 times greater for Asian women than for White women [24]. The rates of stillbirth, neonatal mortality, preterm birth and fetal growth restriction are all significantly greater for babies born to Black and Asian women, and these disparities are compounded by socioeconomic differences [25, 26].

The reasons for these differences are still not well understood. Some co-morbidities, including hypertension and diabetes, disproportionately affect Black or South Asian women. Other known risk factors, such as smoking, are greater in White communities [27]. Health inequalities have complex multifactorial causes. Encompassing more than an individual’s health status or behaviours they have well-documented roots in societal inequalities as well as differential access to and experience of health services [28, 29]. Inequities in maternity care have been increasingly highlighted by women from ethnic minorities, with accounts of systemic racism, discrimination and culturally insensitive care adversely affecting experiences as well as clinical outcomes [29–36]. Evidence from midwifery-led continuity models shows that women from ethnic minorities report better experiences and are enabled to make choices, but such care is the exception not the norm [23, 37].

The growing spotlight on ethnic inequalities in maternity care has prompted calls for more research to understand its causes [27, 33, 38]. It is unclear what role birthplace may play in the differential clinical outcomes and experiences of ethnic minorities. There is global recognition that quality maternity care should be woman-centred and holistic, offering interventions at the right time – neither too soon, nor too late [39, 40]. Midwife-led settings offer care that is particularly suitable for women with uncomplicated pregnancies. Obstetric-led care is appropriate for women with medical or obstetric complexities who may benefit from additional maternal or fetal monitoring or for women who want regional analgesia. High quality antenatal conversations that support informed decision-making about place of birth may enable more women who might benefit from midwife-led care to choose it.

Understanding to what extent and why women from ethnic minority communities do not access different places of birth can therefore help address inequities, improve choices and promote optimal health for women and babies. A mixed-methods study was designed to investigate the association between ethnicity and place of birth in a London NHS Trust.

## Methods

### Design

**Fig. 1:**
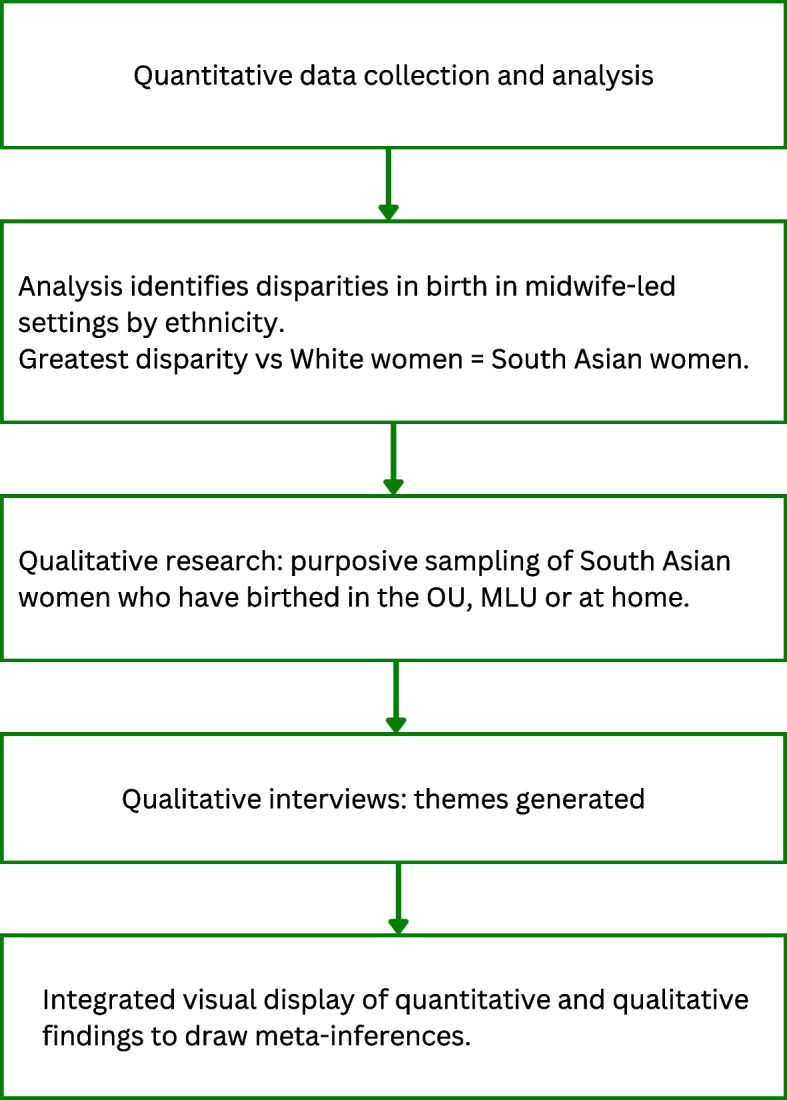
Steps in the mixed-methods sequential explanatory design

A mixed-methods sequential explanatory design [41] sought to investigate the association between place of birth and ethnicity. This involved, first, quantitative exploration of the associations between ethnicity and place of birth; second, qualitative exploration of the choices of the ethnic group with the greatest evidenced disparities compared to White women; and third, the development of an integrated visual display [42] drawing simultaneously on these quantitative and qualitative findings (see Fig. 1).

### Setting

The NHS Trust serves an urban population; 18% of its residents identify as Asian or Asian British – almost double the national average [43]. The area benefits from higher-than-average levels of income, employment and health [44–46]. In 2022–23, the Trust provided maternity care to more than 4600 women. They have the choice to give birth in the OU, the alongside MLU or at home.

### Quantitative methods

Anonymised birth data was collected for the period 2014–2023, including information about selected maternal and birth characteristics. Collection of ethnicity data at the Trust prior to 2014 was not of sufficient quality for inclusion. Births whose default location was the OU – planned Caesareans, Caesareans before the onset of labour, pre-term and multiple births – were excluded from analysis.

Ethnic categories, which are predefined by the Office for National Statistics, have limitations; they group people with varying identities and cultures. It has been argued that creating broad classifications is a form of systemic discrimination [31] and that collective terminology should only be used when there is legitimate justification[33]. Widespread evidence of incomplete or inaccurate data about the ethnicity of maternity service users raises questions about the validity of research findings as well as processes involved in data collection [27, 47].

In view of very small numbers in some categories, these were collapsed into five ethnic groups for the purposes of analysis and to reduce misclassification bias (Table 1) [27, 48]. This is not to suggest that being “South Asian” or “Black” confers homogeneity of beliefs, values and choices. However, the evidence suggests that people from these categories suffer worse clinical outcomes and experiences compared to White women [24–27, 31, 49]. The Trust is located in an area with a substantial population originating from Southeast Asia. “Asian-other” was therefore kept as a distinct group from Other and South Asian. Almost a fifth of births were missing ethnic classification. The absence of recorded ethnicity may relate to experiences of care, therefore we included these births in the analysis.

**Table 1:** Maternal ethnicity at an urban NHS Trust 2014–2023 (*n*=35,387)

**Ethnic classifications used by the Trust (ONS definitions)**	**Ethnic classifications used for statistical analysis**	**n**	**%**
Asian or Asian British – Any other Asian background	Asian - other	1795	5.1
Asian or Asian British – Bangladeshi	South Asian	113	0.3
Asian or Asian British – Indian	South Asian	653	1.8
Asian or Asian British – Pakistani	South Asian	487	1.4
Black or Black British – African	Black	345	1.0
Black or Black British – Any other Black background	Black	207	0.6
Black or Black British – Caribbean	Black	130	0.4
Mixed – Any other mixed background	Other	259	0.7
Mixed – White and Asian	Other	157	0.4
Mixed – White and Black African	Other	75	0.2
Mixed – White and Black Caribbean	Other	118	0.3
Other Ethnic Groups – Any other ethnic group	Other	2326	6.6
Other Ethnic Groups – Chinese	Asian – other	507	1.4
White – Any other white background	White	7164	20.2
White – British	White	14,224	40.2
White - Irish	White	287	0.8
Not stated	Ethnicity unknown	6540	18.5

All percentages rounded to 1 decimal place

Place of birth was categorised into four groups: the OU, the alongside MLU (also known as the Birth Centre), planned homebirth, and unplanned homebirth or other—which included births at home without a health professional present and births in transit or in locations outside the OU, MLU or home.

Statistical analysis was carried out using STATA software version 15.1. A 5% significance level was adopted for all analyses. Chi-squared tests of independence and tests of proportion examined the relationships between maternal ethnicity and selected characteristics. Unadjusted odds ratios were calculated for birthplace by ethnicity. Multivariable logistic regression modelling was used to adjust for potential confounders. These were chosen based on existing evidence that they affect place of birth. Not all potential confounders were available, including socioeconomic data and previous obstetric or perinatal complications, such as postpartum haemorrhage, blood transfusion or admission to the neonatal unit. Maternal age and BMI were categorised into binary groups, as national guidance suggests obstetric-led care for women aged 40 or more and for women with a BMI greater than 35 [1, 2].

### Qualitative methods

Quantitative analysis revealed disparities were greatest between White and South Asian women in place of birth. To explore these, semi-structured interviews were undertaken with South Asian women who had given birth in the OU, the alongside MLU or at home within the last three years under the care of the Trust. Participants were eligible if their heritage was South Asian (for the purposes of this study: Indian, Pakistani, Bangladeshi or Sri Lankan), had not planned a Caesarean birth, and did not have risk factors that would ordinarily exclude them from midwife-led settings according to national or local guidance. Interpreter services were not available, so potential participants were only eligible if they could communicate in English.

### Recruitment

Social media posts on the Trust’s maternity accounts generated initial participants. To ensure each birth setting was represented, additional participants were recruited purposively, by selecting them from the anonymised dataset using a random number generator. Direct contact was made following screening for eligibility. To reduce bias, the intention was to exclude women if the primary researcher (FR) had previously provided them with any clinical care. However, when recruiting women who had planned homebirth, the sample population was so small that this was unavoidable, given the lead researcher’s previous role as a homebirth midwife within the Trust. Women were therefore deemed eligible if the researcher had provided minimal care that was unlikely to have affected birthplace choice. Ten women agreed to be interviewed about a combined total of 17 births, after which saturation of data was achieved.

### Data collection and analysis

Interviews ranged in length from 30 to 55 min. Women were given a choice of interview method: four were interviewed face-to face, one by telephone and five online. Questions were semi-structured, with women encouraged to give views on their decision-making and why they felt White women were more likely to access midwife-led settings. All interviews were recorded, transcribed and coded by the lead researcher using NVivo software to identify themes and sub-themes. Thematic analysis was used to draw meaning from and capture the richness of the data [50].

### Terminology

All the qualitative study participants identified themselves as women. We have chosen to use the words “woman” or “women” throughout, whilst acknowledging that not all people who give birth identify as women. We have described specific ethnic groups where possible. Reference to other studies may necessitate more generic descriptions – such as “women from ethnic minority communities”.

### Positionality

The authors variously have backgrounds in midwifery practice, midwifery research and epidemiology. Ethnicity amongst authors include White British, White European and British Pakistani. Several of us have chosen to practice in midwife-led settings in urban, ethnically diverse populations. Previous and ongoing research interests include access to and use of midwife-led birth settings. These combined perspectives will have contributed to the framing, focus and conclusions of this study.

## Results

### Quantitative results

**Fig. 2:**
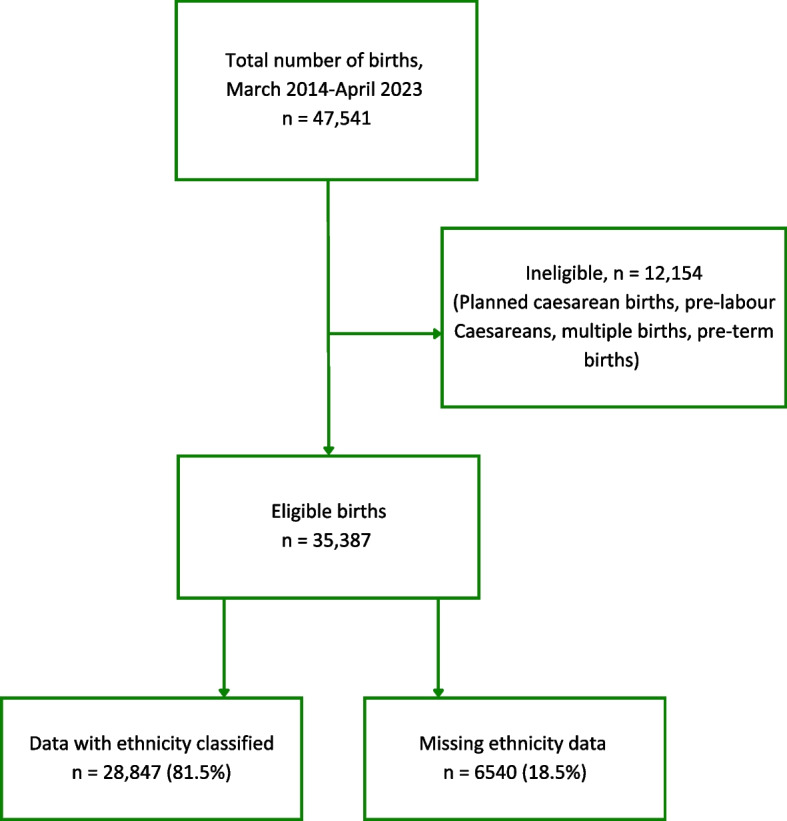
Flowchart of birth data

Between March 2014 and April 2023 there were 47,541 births to women booked at the Trust. The data for 35,387 births was included for analysis, after excluding planned Caesareans, pre-labour Caesareans, multiple and pre-term births (Fig. 2). More than 6500 births had no ethnic classification but were included in the analysis.

Table 2 shows a comparison of selected characteristics by ethnicity. Most women giving birth were classified as White (61.3%), with 6.5% classified as Asian-other, 3.5% South Asian, 1.9% Black, and 8.3% classified as Other. More than 18% of births had no maternal ethnic classification. Statistical analysis demonstrated significant variation across ethnic groups by parity, maternal age, BMI, presence of diabetes or hypertensive disorders, previous Caesarean birth and mode of birth.

**Table 2:** Selected characteristics of births at an NHS Trust by ethnicity, 2014 – 2023

Maternal ethnicity														
	**All**		**White**		**Asian – other**		**South Asian**		**Black**		**Other**		**Ethnicity** **unknown**	
	n	%	n	%	n	%	n	%	n	%	n	%	n	%
**Births**	35,387	100	21,675		2302		1253		682		2935		6540	
**Parity**														
Nulliparous	18,730	53.8	11,425	52.7	1205	52.3	528	42.1	279	40.9	1672	57.0	3621	60.7
Multiparous *X*^*2*^(5)= 252.47, p<0.0001	16,077	46.2	10,245	47.3	1097	47.7	725	57.9	403	59.1	1263	43.0	2344	39.3
Missing (% of total)	580	(1.6)	5	(0.02)	0		0		0		0		575	(9.0)
**Maternal age**														
<30 years	8318	23.5	4924	22.7	575	25.0	358	28.6	238	34.9	832	28.4	1391	21.3
30–39 years	24,686	69.8	15,246	70.4	1596	69.4	830	66.2	395	57.9	1930	65.8	4689	71.7
≥40 years *X*^*2*^(10)= 143.86, p<0.0001	2371	6.7	1496	6.9	128	5.6	65	5.2	49	7.2	173	5.9	460	7.0
Missing (% of total)	12	(0.03)	9	(0.04)	3	(0.13)	0		0		0		0	
**BMI**														
<35	31,560	96.5	19,651	96.3	2125	97.4	1154	96.8	578	91.6	2657	97.0	5395	97.1
≥35 *X*^*2*^(5)= 61.52, p<0.0001	1137	3.5	749	3.7	57	2.6	38	3.2	53	8.4	82	3.0	158	2.9
Missing (% of total)	2690	(7.6)	1275	(5.9)	120	(5.2)	61	(4.9)	51	(7.5)	196	(6.7)	987	(15.1)
Diabetes* *X*^*2*^(5)= 502.08, p<0.0001	2311	6.5	1177	5.4	330	14.3	174	13.9	65	9.5	288	9.8	277	4.2
Hypertensive disorders** *X*^*2*^(5)= 66.04, p<0.0001	2066	5.8	1394	6.4	114	5.0	56	4.5	59	8.7	173	5.9	270	4.1
**Previous Caesarean** *X*^*2*^(5)= 60.66, p<0.0001	1423	5.9	772	4.2	118	6.0	78	7.5	51	8.5	141	5.2	263	4.3
Missing (% of total)	4471	(12.6)	3150	(14.5)	322	(14.0)	215	(17.2)	83	(12.2)	238	(8.1)	463	(7.1)
**Labour onset**														
Spontaneous	24,000	67.8	14,691	67.8	1614	70.2	848	67.7	453	66.4	2019	68.8	4375	66.9
Induced *X*^*2*^(5)= 10.60, p=0.06	11,380	32.2	6981	32.2	684	29.8	405	32.3	229	33.6	916	31.2	2165	33.1
Missing (% of total)	7	(0.02)	3	(0.01)	4	(0.17)	0		0		0		0	
**Mode of birth**														
Spontaneous vaginal	23,561	66.6	14,677	67.7	1472	63.9	835	66.6	490	72.0	1912	65.1	4175	63.8
Assisted vaginal	6697	18.9	4070	18.8	408	17.7	235	18.8	69	10.1	576	19.6	1339	20.5
Caesarean *X*^*2*^(10)= 108.12, p<0.0001	5125	14.5	2925	13.5	422	18.3	183	14.6	122	17.9	447	15.2	1026	15.7
Missing (% of total)	4	(0.01)	3	(0.01)	0		0		1	(0.15)	0		0	

All percentages rounded to 1 decimal place. * pre-existing and gestational diabetes. ** includes pre-existing and gestational hypertension, pre-eclampsia and HELLP

South Asian and Black women were more likely to be multiparous than White women (57.9% and 59.1% vs 47.3% respectively, *p* < 0.001). The mean age of all women giving birth was 32.6 years, with the youngest aged 14 and the oldest aged 50. White women were older than all other ethnicities. Black women were more than twice as likely to be obese than women of other ethnicities (8.4% vs 3.5%, *p* < 0.001), while Asian-other women had significantly lower BMIs than White women (*p* = 0.01).

Pre-existing or gestational diabetes was identified in 6.5% of all women. Pre-existing or gestational hypertensive disorders were recorded in 5.8% of women. White women were less likely than all other ethnic groups to have or develop diabetes (5.4%) with Asian-other and South Asian women more than twice as likely to have or develop the condition (14.3% and 13.9% respectively, *p* < 0.001). Black women had the highest rates of hypertensive disorders (8.7%) and South Asian women had the lowest (4.5%). Just under 6% of all women had had a previous Caesarean birth – most of the population who had previously given birth by Caesarean were excluded from analysis as they planned a Caesarean for their subsequent birth. White women were less likely to have previously given birth by Caesarean compared to all other ethnicities, with South Asian and Black women almost twice as likely to have had a Caesarean birth (7.5% and 8.5% vs 4.2% respectively, *p* < 0.001).

Two thirds of women had a spontaneous onset of labour. The proportion of induced labours was similar amongst women from White, South Asian, Black, Other and unknown ethnicities. Asian-other women had slightly fewer inductions (*p* = 0.019). Two thirds of women – whether labour was induced or began spontaneously – had a spontaneous vaginal birth. White women were less likely to have an intrapartum Caesarean (13.5%) than all other ethnicities. Black women had the highest rate of spontaneous vaginal birth (72%) while Asian-other women had the highest rate of intrapartum Caesarean (18.3%).

### Place of birth

Most women gave birth in the obstetric unit (73.7%), 24.1% in the midwife-led unit and 2.3% at home (Table 3). Less than 1 percent of women gave birth in a different location within the hospital, in transit or at home but without a health professional present. These births were not included in statistical analysis of birthplace; there was no significant difference in distribution across ethnic groups or births missing ethnic classification (*p* = 0.261).

**Table 3:** Place of birth by maternal ethnicity at one NHS Trust 2014–2023

Maternal ethnicity														
	**All**		**White**		**Asian – other**		**South Asian**		**Black**		**Other**		**Ethnicity unknown**	
	n	%	n	%	n	%	n	%	n	%	n	%	n	%
*Place of birth*	35,074	100	21,465		2286		1247		674		2913		6489	
**Delivery Suite (OU)**	25,844	73.7	15,554	72.5	1755	76.8	990	79.4	511	75.8	2218	76.1	4816	74.2
**Birth Centre (MLU)**	8438	24.1	5329	24.8	515	22.5	247	19.8	143	21.2	644	22.1	1560	24.0
**Planned Homebirth**	792	2.3	582	2.7	16	0.7	10	0.8	20	3.0	51	1.8	113	1.7

Analysis of birthplace excludes unplanned homebirths and births in locations outside the OU, MLU or home (*n* = 311, 0.9% of eligible births). Place of birth was missing for 2 births

All percentages rounded to 1 decimal place

*X*^*2*^(10) = 107.91, *p* < 0.0001

Place of birth varied according to ethnicity (see Tables 3, 4, 5 and 6). The proportion of births in midwife-led settings was highest for White women (27.5%), followed by Black (24.2%), Other ethnicity (23.9%), Asian-other (23.2%) and South Asian women (20.6%). South Asian women were much less likely to birth in a midwife-led setting after adjusting for parity, maternal age, BMI, previous Caesarean, presence of diabetes or hypertensive disorders and onset of labour (aOR 0.61, 95% CI 0.51–0.73, *p* < 0.001). Following adjustment for confounders, the difference also remained significant for Asian-other and Other women, but not for Black women or those with no ethnic classification (Table 4). However, the number of Black women in the population was too low to detect significant differences.

**Table 4:** The association between maternal ethnicity and birth in a midwife-led setting at one NHS Trust 2014–2023 (*n* = 35,074)

	**Midwife-led setting** **(MLU and planned homebirth)**		**OU (Delivery Suite)**		**Unadjusted OR (95% CI)**		***p*-value**	**Adjusted OR (95% CI)**		***p*-value**
*Ethnicity*	**n**	**%**	**n **	**%**						
White	5911	27.5	15,554	72.5	1.0		-	1.0		-
Asian – other	531	23.2	1842	76.8	0.80 (0.72–0.88)		<0.0001	0.70 (0.61–0.79)		<0.0001
South Asian	257	20.6	1063	79.4	0.68 (0.59–0.79)		<0.0001	0.611 (0.51–0.73)		<0.0001
Black	163	24.2	555	75.8	0.84 (0.70–1.00)		0.055	0.85 (0.67–1.06)		0.154
Other	695	23.9	2358	76.1	0.82 (0.75–0.90)		<0.0001	0.82 (0.73–0.91)		<0.0001
Ethnicity unknown	1673	25.8	5058	74.2	0.91 (0.86–0.97)		0.005	0.98 (0.90–1.06)		0.616

All percentages rounded to 1 decimal place

*Unadjusted X*^*2*^(5) = 61.80, p < 0.0001

*Adjusted X*^*2*^(12) = 7314.11, *p* < 0.0001

The biggest difference in birthplace by ethnicity was observed for planned homebirth, with 2.7% of White women giving birth at home, compared to 0.7% of Asian-other women and 0.8% of South Asian women (Table 5). After adjusting for confounders, the difference remained significant for Asian-other (aOR 0.23, 95% CI 0.13–0.41, *p* < 0.001), South Asian (aOR 0.34, 95% CI 0.18–0.64, *p* = 0.001) and Other women (aOR 0.67, 95% CI 0.49–0.92, *p* = 0.014).

**Table 5:** The association between maternal ethnicity and homebirth at one NHS Trust 2014–2023 (*n* = 35,074)

	Homebirth		Hospital birth (OU and MLU)		Unadjusted OR (95% CI)	*p*-value	Adjusted OR (95% CI)	*p*-value
*Ethnicity*	n	**%**	**n**	**%**				
White	582	2.7	20,883	97.3	1.0	-	1.0	-
Asian – other	16	0.7	2270	99.3	0.25 (0.15–0.42)	<0.0001	0.23 (0.13–0.41)	<0.0001
South Asian	10	0.8	1237	99.2	0.29 (0.15–0.54)	< 0.0001	0.34 (0.18–0.64)	0.001
Black	20	3.0	654	97.0	1.10 (0.70–1.73)	0.688	1.06 (0.63–1.77)	0.834
Other	51	1.8	2862	98.3	0.64 (0.48–0.85)	0.002	0.67 (0.49–0.92)	0.014
Ethnicity unknown	113	1.7	6376	98.3	0.64 (0.52–0.78)	< 0.0001	0.60 (0.47–0.77)	< 0.0001

*All percentages rounded to 1 decimal place*

*Unadjusted X*^*2*^(5) = 82.59, *p* < 0.0001

*Adjusted X*^*2*^(10) = 391.47, *p* < 0.0001

When comparing the proportion of births in the MLU with births in the OU (Table 6), White women were more likely to birth in the MLU (25.5%) than South Asian (20%) and Asian-other women (22.7%), a difference which remained significant after adjusting for confounders (aOR 0.64, 95% CI 0.54–0.77, *p* = 0.015 and aOR 0.75, 95% CI 0.66–0.85, *p* = 0.017, respectively).

**Table 6:** The association between maternal ethnicity and birth in a midwife-led unit at one NHS Trust 2014–2023 (*n* = 34,282)

	**MLU**		**OU (Delivery Suite)**		**Unadjusted OR (95% CI)**	*p*-value	Adjusted OR (95% CI)	*p*-value
*Ethnicity*	**n**	**%**	**n**	**%**				
White	5329	25.5	15,554	74.5	1.0	-	1.0	-
Asian – other	515	22.7	1755	77.3	0.86 (0.77–0.95)	< 0.0001	0.75 (0.66–0.85)	0.017
South Asian	247	20.0	990	80.0	0.73 (0.63–0.84)	< 0.0001	0.64 (0.54–0.77)	0.015
Black	143	21.9	511	78.1	0.82 (0.68–0.99)	0.147	0.84 (0.66–1.06)	0.565
Other	644	22.5	2218	77.5	0.85 (0.77–0.93)	0.002	0.84 (0.75–0.94)	0.183
Ethnicity unknown	1560	24.5	4816	75.5	0.95 (0.89–1.01)	0.717	1.02 (0.93–1.10)	0.792

All percentages rounded to 1 decimal place

*Unadjusted X*^*2*^(5) = 38.66, *p* < 0.0001

*Adjusted X*^*2*^(12) = 6568.95, *p* < 0.0001

### Qualitative findings

Table 7 provides details of interview participants’ characteristics.

**Table 7:** Participant characteristics (*n* = 10)

**Age at most recent birth**	
20–29	3
30–39	6
40–49	1
**Ethnicity**	
Pakistani	4
Indian	3
Sri Lankan	3
**Country of birth**	
UK	5
Pakistan	3
India	1
Other	1
**Religion**	
Muslim	4
Hindu	4
Sikh	1
None	1
**Parity before most recent birth**	
0	5
1	4
2 +	1
**Intended place of birth (including all previous births,** ***n*** **= 17)**	
Home	5
MLU	5
OU	7
**Actual place of birth**	
Home	5
MLU	3
OU	9

Six key themes were generated following thematic analysis of the interviews:

Cultural assumptions – birth takes place in hospital with doctors.

Going against the cultural norm.

Choosing midwife-led care to feel safe.

Past experiences influence future choices.

Sources of knowledge – from midwives to Instagram.

Reducing barriers to midwife-led care – education and visibility.

### Cultural assumptions—birth takes place in hospital with doctors

All the women referred to an obstetric-led setting as their original default assumption for place of birth, with many acknowledging that their cultural upbringing had influenced this.

“I always used to feel, oh my god, delivering a baby without doctors is ancient…we live in a modern world, going to hospital, having doctors around you…So it definitely has an influence of where I come from” – (009, planned OU 1st baby, planned homebirth 2nd baby).

Alongside this assumption was a perception of safety and trust in doctor-led care and a parallel uncertainty about midwives and midwife-led care. Participants spoke about a reverence for doctors in their culture and many expressed feeling reassured by the availability of medical interventions. Several remarked on a societal or cultural perception that midwives were associated with outdated, village practices. Those views were more explicitly made, but not exclusively, by the participants who had lived most of their lives in South Asia.

“It’s just a sense of security that I felt if I go to the Labour ward…it would be more relaxed and less stressed…I thought I would have surgeons or consultants, if required they would be there…In Pakistan…a birth would never be entirely, you know, supervised by a nurse. There's no concept of midwife or anything like that.” – (010, planned 1st baby in OU).

Just as a birth without doctors was considered unusual, so too was using a birthing pool – a choice most commonly associated with midwife-led settings. Several women identified a lack of knowledge about birthing pools in their communities, which they felt would deter women from opting to use them. Although all the women interviewed were informed during their pregnancies that pools were an option, this cultural unfamiliarity with and suspicion of the concept of waterbirth and its potential benefits influenced many in their initial decision making.

“I was thinking that I don't want to be in water giving birth, you know, I have not seen much of that in where I come from.” – (009, planned OU 1st baby, planned home waterbirth 2nd baby).

“I spoke to my mum about it and in our religion, I don’t know if it’s religion or maybe in our culture, she was like, you can use that as a pain relief but when it’s time to give birth just make sure that you’re on the bed and he’s on land rather than in water.” – (006, planned MLU 1st baby, planned OU 2nd baby).

The experiences and advice relayed to women from mothers, sisters and aunts appeared to be given significant weight. Steeped in those influential narratives, most women assumed they would have a vaginal birth. Many also assumed, at least at the start of their pregnancy, that doctors would need to be involved by default. Some spoke of surprise, which was reinforced by comments from relatives, at the prospect of perhaps not meeting a doctor at any point during their pregnancy and birth. These familial voices of concern about birth as an event that required medical supervision was dominant for many in influencing their choice of birth setting.

### Going against the cultural norm

For those women that chose midwife-led births, most recognised that they were doing something outside the cultural norm. Becoming pregnant sparked an openness to influences beyond their communities and a willingness to listen to other voices. Many of them displayed a strong sense of personal autonomy and independence.

“Once I found out about the different options, I thought I’m going against what people would traditionally have opted to do…it felt quite empowering” – (001, planned MLU birth 1st baby).

Explaining their choices to family members was met with a variety of responses, from confusion to alarm. Women described concerns from relatives about safety, and unease that they were turning their backs on medical help. The decision to make alternative choices brought with it a sense of responsibility, and the feeling that if anything went wrong, they would be blamed for making a rash or unwise decision.

“A lot of my aunties when they found out I had a homebirth. Very shocked. ‘Why did you choose that? You’ve got no doctors there.’” – (003, planned MLU 1st baby, planned homebirth 2nd baby).

“I had to say it’s not because I’m anti-hospital or I’m anti-medication.” – (004, planned homebirth 1st baby).

Anticipating negative reactions, some women decided not to share their choices for a midwife-led birth with the wider family, even those who decided to opt for care in the alongside MLU, located on the same floor as the obstetric unit. There was a sense of wanting to protect themselves from potential criticism, to not have to explain or justify decisions. Others felt more able to defend their choice when challenged, citing evidence they had researched themselves of lower intervention rates.

“There was some concern that it was a riskier choice. My response to that was…actually the research suggests that the option that you feel is the most secure results in increased C-sections, increased use of forceps, ventouse…I think they quickly realised that it wasn’t really their call. So then they backed down.” – (008, planned MLU 1st baby, planned homebirth 2nd baby).

Those that chose a homebirth acknowledged that their decision would have been different if they lived in a multi-generational household, an arrangement common to some South Asian communities. They spoke of the need for privacy, of birth as something that would not traditionally involve men. All the women interviewed who did choose homebirth lived only with their husband. Partner support was viewed as critical in influencing birthplace decisions.

“I said, ‘Oh what do you think about homebirth?’ he kind of immediately shut me down and said if anything were to happen, you would blame yourself’…that was kind of the end of it” – (002, planned MLU 1st baby).

“Most of the time men [from South Asian cultures] don’t really get involved in a lot of stuff [to do with childbirth] and to have a baby at home is just not common, but because he was very interested naturally made me think, oh if he’s okay with it, maybe we should look into it.” – (004, planned homebirth 1st baby).

### Choosing midwife-led care to feel safe

Several women spoke of feeling safer—physically, psychologically and culturally—in a midwife-led setting. They mentioned benefits like knowing their midwife, being in a more holistic and less clinical environment, and avoiding medical interventions. The MLU offered the ideal birth setting for some, as they felt a sense of security provided by the knowledge that doctors were close by.

“What’s the downside? The doctors aren’t going to say ‘Sorry, we’re not going to see you because you opted for a birth centre birth.’ It just felt like a total no brainer.” – (008, planned MLU 1st baby, planned homebirth 2nd baby).

For others, who chose a homebirth, the sense of psychological and cultural safety came from knowing they were less likely to encounter male caregivers, could enjoy complete privacy and could form relationships with a trusted team of midwives who they did not feel would judge them. One woman described an incident of being spoken to in the hospital as if she did not understand English, which reinforced her birthplace decision.

“It kind of ticked all my personal boxes knowing that I could be at home, not have to worry about what I’m wearing…on that level it was just perfect… the comfort of knowing who you’ve got [looking after you], as someone who is obviously Muslim, who covers [with a hijab]….I knew who I was going to be around…and knowing that [the midwife], you know, we have a relationship or a connection made me feel very comfortable.” – (004, planned homebirth 1st baby).

For another woman, her decision not to give birth again in hospital related to an encounter with a member of staff that made her feel discriminated against. Her anxiety that she might experience such attitudes again was a significant driver in seeking the sanctuary of her own home, and care from people she could get to know and trust.

“Being told that I’m not dealing with pain as well as others was not a good thing, and it’s not just me, like I know that from very close friends who are also Indians we’ve heard that, almost suggesting that the pain threshold in Asian women is lower than others…I just need to work with people who get me and who do not judge me …I think that’s why I thought I need to explore the home birth option. What I didn’t want was the hospital experience.” – (009, planned OU 1st baby, planned homebirth 2nd baby).

The chance to build a relationship with one person or team was frequently cited by those opting for a homebirth. The benefits were felt particularly by those who had experienced a more disjointed approach in a previous pregnancy. Women described the contrast between routine care, which often involved a different midwife at each appointment with limited time versus continuity of care in their home with the same midwife who they felt wanted to know and understand them at a deeper level.

“Amazing. Like really, really amazing … It was like, you know, having a friend who's just helping you do this. It just was yeah, one of the most positive things I've experienced in my life.” – (009, planned OU 1st baby, planned homebirth 2nd baby).

### Past experiences influence future choices

Women’s experiences of their birth significantly influenced their subsequent birthplace choice or thoughts about future potential pregnancies. When birth experiences were positive, there was a tendency to want to repeat the experience, wherever the birth took place. For those who planned birth in the OU or at home, if birth had gone as expected, this reinforced their views about their chosen birthplace as the safest place to be. Several women who had planned birth in the MLU for their first child—whether they transferred to the OU during labour or remained there—expressed a wish to choose a homebirth for their subsequent birth, expressing feelings of greater confidence in themselves and their bodies.

“I can almost pinpoint those little moments that happened [in labour] that make me think ‘oh no’… if I just stayed at home I think I would have been fine…so if we do have another baby, I would like to try for a homebirth. Definitely.” – (002, planned MLU 1st baby).

### Sources of knowledge – from midwives to Instagram

The information women gained from their midwives about birthplace appeared to depend on the type of care they received and if they themselves asked questions. Those who had continuity of care described detailed information sharing, whereas those who saw different midwives at each appointment described more limited exploration of choices. Time constraints were mentioned by several women as a barrier to having more in-depth discussions with their midwives, with women perceiving their midwife’s focus to be the completion of certain tasks and checklists.

“It was literally a quick question, you know ‘Where do you want to give birth?’ and I was like hospital, and that was it. There wasn’t much further information like, maybe you could consider homebirth, you know…it was only until my friend told me, then it opened my eyes. If she had never had a homebirth, I probably would have had [my son] at the hospital.” – (003, planned MLU 1st baby, planned homebirth 2nd baby).

One woman, who arrived in the UK towards the end of her first pregnancy, described minimal information sharing about what to expect. She wasn’t offered any antenatal classes and sought out her own knowledge online. When she went into labour, she called an ambulance, not appearing to understand it was an emergency service. The idea that she might have had different options about where to give birth did not seem to occur to her, nor to have been offered or explained.

“During my first baby I didn’t make choice. I wasn’t aware of everything because it was my first baby…you don’t have any idea what is labour, what is contractions, nothing.” – (007 planned OU births).

Most of the women who planned midwife-led births cited other sources of information beyond that provided by their midwife which were key in questioning their assumptions about birth. Conversations with friends or family members who had experienced births in midwife-led settings were particularly influential for several women, particularly if those friends were from the same ethnic community or religious background. Three of the five women who planned homebirths referenced discussions with key figures in their family or community who had given them the confidence to choose it for themselves.

“I probably thought oh [homebirth] isn’t for me, but I think hearing from someone of the same…religious views, the same age group, made me think oh ok maybe it isn’t just for people that don’t like hospitals.” – (004, planned homebirth 1st baby).

For others, external sources like attending yoga, antenatal or hypnobirthing classes or following birth-related accounts on social media were just as significant. Women spoke of being inspired to confront their own assumptions about birth as a medical event. Those courses or social media accounts were more likely to be dominated by women from White communities; several participants noted they might be the only South Asian woman present at a class or yoga session. Acquiring external knowledge and being exposed to alternative viewpoints appeared to validate their decisions—and give them courage to challenge pushback from within their own communities about their birth choices.

“I followed on social media – Instagram specifically – a lot of parents, mums, who are influencers, who aren’t…Asian ethnicity, and when they were getting pregnant…they were doing things like hypnobirthing, yoga…so for me it was almost normalised….I saw it so often on social media that when I got pregnant I was like of course I’m going to do that” – (002, planned MLU 1st baby).

### Reducing barriers to midwife-led care – education and visibility

When interviewees were informed about the disparities in access to midwife-led settings between White and South Asian women, several common solutions emerged. All women mentioned the need for more education about place of birth, with some suggesting information targeted directly at women of their ethnicity. There was a sense from some that the nuances of their own culture or religion were not well understood by those outside their communities; they felt that hearing from other mothers who looked like them—at face-to-face classes or shared in videos online—would carry more weight and open more people’s eyes to midwife-led settings as a safe choice.

“If someone who isn’t Muslim or isn’t from an ethnic background just said ‘Oh I had a great time’, they might not talk about things like having a female [midwife] or having privacy. They won’t because it’s not part of their life.” – (004, planned homebirth 1st baby).

“I’ve only ever experienced white babies, pictures and stuff like that…[seeing women who look like her] just helps the mother feel a bit more seen” – (006, planned MLU 1st baby).

There was a sense that a standardised conversation, a “one size fits all” approach, about birthplace did not work. Some participants expressed a need for midwives to take extra steps with women from ethnic minorities to expose them to the potential benefits of midwife-led settings, given their cultural assumptions about birth as a doctor-led event.

“The safe option [for South Asian women] feels like picking the doctor side…especially if you have voices of concern who you’re used to listening to like elders, which is big in our culture” – (008, planned MLU 1st baby, planned homebirth 2nd baby).

These extra steps meant not just sharing statistics and evidence, but relaying stories; stories of women like them who had experienced birth in the MLU or at home, to bring alive a theoretical choice and make it a reality.

### Integration of quantitative results and qualitative findings

**Fig. 3:**
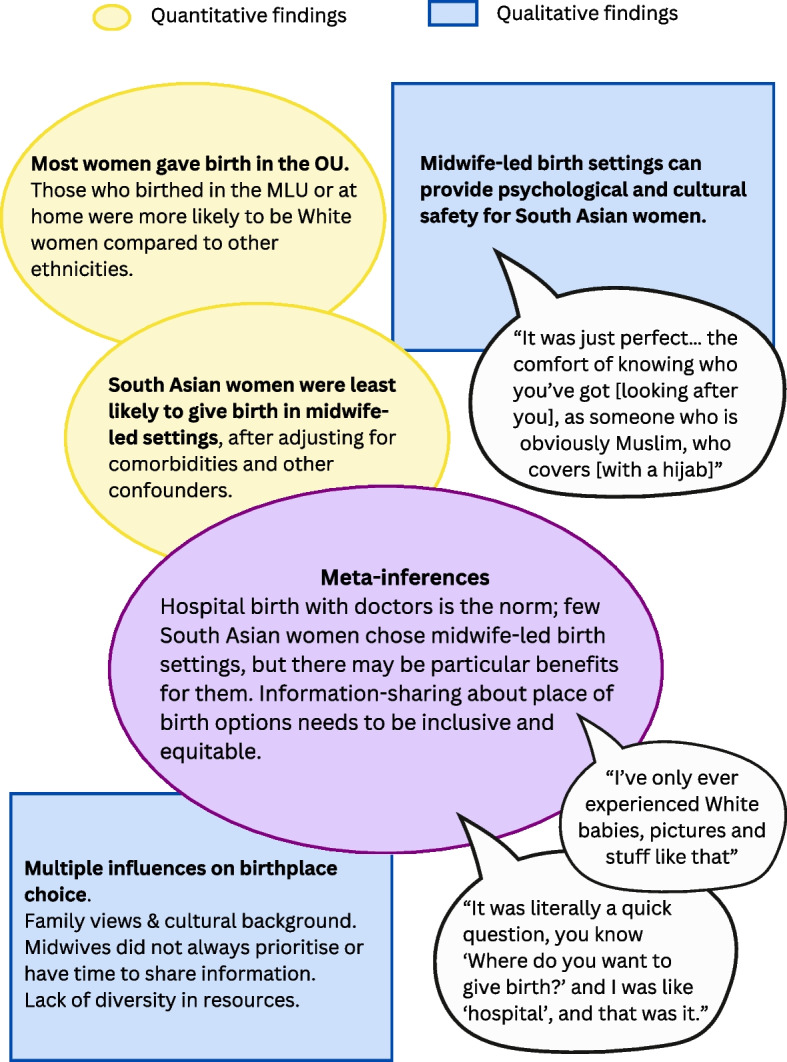
Joint display of quantitative and qualitative findings

Figure 3 illustrates the quantitative and qualitative findings in a joint visual display, to provide explicit integration of the results and development of meta-inferences [42].

## Discussion

There is a significant independent association between ethnicity and place of birth at this NHS Trust. Compared to White women, Asian women are less likely to give birth at home or in the MLU. Interviews with South Asian women suggested a combination of factors behind this. The cultural assumption was that birth should take place in hospital with doctors. Midwives did not always share information about options or challenge this assumption, and this was exacerbated by poor continuity of care and time pressures. When women chose midwife-led settings they were influenced by external factors such as friends who had made similar choices, or they researched options for themselves. Choosing to give birth at home or in the MLU made them feel empowered and safe – physically, psychologically and culturally. Some were met with negative responses from family members, and others chose to conceal their birthplace choices. The need for more education about birthplace was highlighted by all participants, with many suggesting information directly targeted at and representative of women from different ethnic minorities.

Despite national drivers to increase choice of place of birth [1, 3] since the publication of the Birthplace in England study [19], there has been limited quantitative analysis of place of birth by ethnicity in the last decade. Unlike the Birthplace study, this study shows large differences by ethnicity between women birthing in the alongside MLU and the OU, with South Asian women especially being much less likely to have a midwife-led birth. Overall, the findings add to the growing body of evidence demonstrating ethnic disparities across the maternity care spectrum. While inequalities in care and outcomes are sometimes attributed to comorbidities differentially affecting women from ethnic minorities, the differences in this study persisted even after adjusting for those factors.

Many of the themes which emerged from the qualitative research support findings from previous studies about place of birth and decision-making. The assumption that birth is an event that takes place in hospital with doctors appears to be a strongly held belief across sociodemographic groups and has been widely reported in literature [15, 51, 52]. Studies have suggested that White women appear to be more aware of choices while women from ethnic minorities may experience a lack of choice [21, 22, 53–55]. A study of Pakistani migrant women in Wales revealed uncertainty about the role of the midwife [56]. Similarly, for some of the women in this study who were not familiar with the UK maternity system and who chose an OU birth, the very concept of a midwife, let alone a midwife-led birth-setting, was not well understood.

We are aware of only one other qualitative study which explores the decisions of South Asian women to birth outside the OU. Reeve Jones studied British-born Bengali women choosing to give birth in an urban freestanding birth centre [57]. Although none of the participants in this study were Bengali, their narratives are similar. Women felt a need to justify their decision, a burden of responsibility in case anything went wrong and therefore frequently concealed their choice from family members who viewed the OU as safest. Similarly, the women in this study who chose the MLU or homebirth were aware their choices did not fit the cultural norm and talked of negative or confused reactions from family. However, like the Bengali women, they also felt a sense of empowerment in their choice.

Many were influenced by external sources in deciding to birth outside the OU, which chimes with other studies about place of birth decision making [15, 52]. Midwives have been shown to be both facilitators of and a barrier to the decision-making process [15, 58]. Lack of time, poor continuity and not prioritising birthplace discussions were highlighted by several of the women in this study. The benefits of continuity of midwifery care are extensively documented [6, 37] including around decision-making [31, 59] and this was apparent for the women in this study who chose a homebirth. Their decisions were validated by their midwives – which enabled them to push back against any criticisms from friends and family.

Some women called for greater visibility of women who “look like me” in birth images and narratives as a way to encourage use of midwife-led birth settings. Some also called for evidence-sharing that acknowledged cultural differences. This has not been previously explored in research about women’s information needs for choosing their place of birth [15, 52, 58].

A significant finding was the concept of choosing a homebirth as a culturally safe option. A growing body of evidence about the maternity experiences of women from ethnic minorities has demonstrated a disregard for cultural and religious needs [31, 32, 34, 35, 60]. Requests for female care providers, for example, have been turned down, leaving women feeling disempowered [34, 60, 61]. Participants in this study were asked whether they had experienced discriminatory care in a maternity setting. All the women described positive interactions with most midwives and doctors. Several said their ethnicity was not relevant in how they felt treated or spoken to. However, two participants described situations which made them question whether the care or attitudes of the health care professionals were related to their skin colour. Their decision to give birth at home was a way to avoid discrimination, to control their birth environment and the people who entered it, and to form a positive relationship with a supportive midwifery team. A Pakistani woman, who was Muslim, explained that birthing at home meant she did not worry about feeling judged or about whether she might receive care from a male healthcare professional. This example demonstrates the importance of acknowledging women’s wider intersecting identities (such as religion) which can influence their choices and place of birth, and avoiding assumptions based only on ethnicity.

### Strengths

This study adds novel findings about place of birth and ethnicity in the UK, which is an under researched area. It is one of a handful of studies to investigate the birthplace decisions of South Asian women living in the UK. It is the only known qualitative study exploring the motivations of South Asian women who have planned or had a homebirth. It is the only known quantitative analysis of birthplace data by ethnicity since the publication of the Birthplace in England study [19]. Most participants were purposely selected by place of birth from the anonymised dataset which yielded a diverse range of participants by country of origin and background.

### Limitations

The findings of this study are from one NHS Trust and therefore may not be generalisable to the wider maternity population, although it serves a large, ethnically diverse population. Data on some potential confounders were not available, which might have changed the significance and strength of the association between ethnicity and birthplace. However, the characteristics of the women are similar to a recent national maternity audit of ethnic inequalities in more than 1.2 million mothers [27]. This was a retrospective analysis which did not provide information about women’s intended place of birth. It is not possible to assess if this differed significantly by ethnicity from their actual birthplace.

The proportion of births missing ethnic classification of the mother was high. However, only two births were missing information about place of birth. For the main outcome – which was birth in a midwife-led setting – there was no significant difference between White women and women with no ethnic classification. There was also missing data about other maternal characteristics including previous Caesarean and BMI which was differentially distributed by ethnicity. This may have biased results in either direction for those variables.

The “race of researcher effect” is a potential limitation of this study [62–64]. There is no consensus about whether ethnic congruence between researcher and participant is desirable [62–64]. While some sensitive topics may be more easily disclosed to researchers from a similar background [55, 64], participants can be reluctant to share information with people who are part of their community [63, 65] and the researcher as “outsider” is a potential advantage [64]. What is essential is a culturally competent researcher who is able to build trust with participants [63].

### Recommendations

There is a need for better information for women from ethnic minorities about place of birth, including images and narratives that reflect their experiences. Better understanding of midwife-led settings could increase usage [66]. This study has highlighted how homebirth can enable culturally safe care for South Asian women, among whom homebirth levels were disproportionately low, with the benefits of continuity and a familiar, private environment. Birthing in an MLU may have similar advantages, with a philosophy that facilitates autonomy and a woman-centred social model of care [57, 67]. The importance of care that is psychologically and culturally safe cannot be overstated. An overarching focus on physical safety risks side-lining the impact that poor and disrespectful care can have on clinical outcomes and long-term psychological health, and this is especially relevant to ethnic minority communities [24, 31, 49, 68]. Women from ethnic minorities, particularly those with uncomplicated pregnancies, therefore may stand to benefit the most from midwife-led care. Midwives may benefit from training that improves the quality, content and cultural competency of birthplace conversations.

The significant gap in the recording of ethnicity data is not unique to this study [27, 33]. It is vital that maternity services prioritise collection of accurate sociodemographic characteristics [38]. A lack of accurate data risks masking the extent of disparities. Closing the ethnicity data gap will enable a better understanding of differential outcomes – and allow services to be held to account.

This study has exposed existing inequalities in birthplace choices. Further research is warranted to investigate the benefits, access to, and use of midwife-led birth settings by ethnic minority and marginalised communities, including those for whom English is not their first language. A larger quantitative study that accounts for socioeconomic difference would be useful to assess validity. The maternity landscape has changed significantly since the Birthplace in England study was published [19], with efforts to improve choice, continuity and clinical outcomes [1, 3, 69–71] set against a backdrop of midwifery workforce challenges [47], reports of substandard care [72–74], a global pandemic and ethnic disparities across the maternity spectrum. It is an opportune time to look again at birthplace data through an ethnicity lens.

## Conclusion

Choosing where to give birth is a decision whose importance is recognised in UK maternity policy and one that should be available to all women. However, like other aspects of maternity care, there are ethnic inequalities. White women are more likely to give birth in midwife-led settings than other ethnicities in the UK. Cultural factors seem influential, but barriers to choice, such as limited information provision, may disproportionately affect women from ethnic minority communities, who may particularly benefit from midwife-led birth settings. It is important to investigate further why these disparities exist. It is possible that midwives make assumptions about what is culturally acceptable to some ethnic minority communities and therefore do not offer birthplace choices to all women. Women need personalised information about options. Midwives need support in holding high quality conversations. Improving choice of birthplace is a step towards reducing health inequalities and promoting optimal health.

## Data Availability

The dataset used and analysed during the current study is available from the corresponding author on reasonable request.
